# Maxillofacial Prosthesis in Dentofacial Traumas: A Retrospective Clinical Study and Introduction of New Classification Method

**DOI:** 10.1155/2017/8136878

**Published:** 2017-02-28

**Authors:** Edoardo Brauner, Giorgio Pompa, Alessandro Quarato, Sara Jamshir, Francesca De Angelis, Stefano Di Carlo, Valentino Valentini

**Affiliations:** Dipartimento Scienze Odontostomatologiche e Maxillo Facciali, Università degli Studi di Roma La Sapienza Facolta di Medicina e Odontoiatria, Roma, Italy

## Abstract

*Background*. Maxillofacial trauma represents a field of common interest as regards both the maxillofacial surgery and prosthodontics, especially for the functional and aesthetic stomatognathic rehabilitation. This condition necessitates relationship between maxillofacial surgeon and prosthodontist, to achieve the ultimate treatment goal.* Purpose*. The purpose of this study is to make predictable patients outcomes classifying their clinical data, using certain parameters and introducing a new classification method.* Materials and Methods*. We have chosen 7 parameters to classify the entity of the damage of these patients and to make their treatment and their prognosis predictable: number of teeth lost (T1–T4), upper/lower maxilla (U/L), alveolar/basal bone (Alv/B), gingival tissues (G), soft tissues (S), adult/child (a/c), and reconstructed patient (R).* Results and Conclusions*. The multidisciplinary approach and the collaboration between multiple clinical figures are therefore critical for the success of the treatment of these patients. The presence and quantification of above parameters influence the treatment protocol; patients undergo different levels of treatment depending on the measured data. The recognition of certain clinical parameters is fundamental to frame diagnosis and successful treatment planning.

## 1. Introduction

Trauma is the leading cause of death in the first 40 years of life [1]. WHO Statistics indicate that 1 million people die and between 15 and 20 million are injured annually in road traffic accidents [1].

Craniomaxillofacial trauma is relatively common and the vast majority involve concomitant soft tissue injuries [2].

Management of these injuries includes treatment of facial bone fractures, dentoalveolar trauma, and soft tissue injuries, as well as associated injuries [3].

Maxillofacial trauma represents a field of common interest as regards both the maxillofacial surgery and prosthodontics, especially for the functional and aesthetic stomatognathic rehabilitation.

This condition necessitates relationship between maxillofacial surgeon and prosthodontist, to achieve the ultimate treatment goal.

Many variables must be considered during treating traumatized patients: age, sex, sites, etiology, concurrent loss of tissues, associated fractures, treatment modality, complications, and postoperative assessment and follow-up [4].

Epidemiologically most affected individuals are male (M : F 3 : 2) aged between 15 and 40 years [1, 5–8].

Premaxilla is the most affected area with regard to its particular anatomical conditions that predispose to increased exposure trauma.

For the same reason the incidence is doubled in subjects with a maxillary protrusion (2nd class first division).

Fractures of the jaws are frequently associated with dentoalveolar fractures: the association can take place indirectly through occlusion forced to concomitant low kinetic energy trauma (assaults, falls, and sports injuries) or directly as a result of traumatic events at high speed (traffic accidents, gunshot fire).

The most common etiologic agents are represented by road accidents, assaults, falls, blows gunshot, sports injuries, or workplace injuries.

Other risk groups are epileptics, drug addicts, or patients receiving radiotherapy of the jaws.

Such injuries' outcomes represent a big challenge for maxillofacial and oral surgeons and in the end for the prosthodontist because the dentoalveolar defects can reduce the retention and stability of the prosthesis.

Prosthodontist has different treatment options to replace missing soft and hard tissues, including removable dental prostheses.

The options for a prosthetic rehabilitation are either the tooth-supported prosthesis or implant supported overdenture [9].

Concerning the rehabilitation choice between fixed and removable prosthesis, technical considerations are important, such as implant position, aesthetic result, or psychological considerations like acceptability of a removable prosthesis, and not less important, the economic possibilities [10]. However each rehabilitation proposal must be fitted on patient necessity and request.

Rehabilitation should be planned, when possible, before surgical treatment, in order to cooperate with the maxillofacial surgeon in choosing the most appropriate restorative treatment [11].

The purpose of this study is to make predictable patients outcomes classifying their clinical data, using certain parameters and introducing a new classification method.

## 2. Materials and Methods

This study is based on a retrospective review performed on 50 patients outcomes of trauma treated in Implantoprosthesis Unit of Head and Neck Department in “La Sapienza” University of Rome, Policlinico “Umberto I” (Table 1).

All the patients were rehabilitated in the period between 2008 and 2015 and received bone reconstruction, implant positioning, and fixed prosthesis. All the patients are in follow-up. All patients have inserted prosthesis at least six months before.

We have chosen 7 parameters to classify the entity of the damage of these patients and to make their treatment and their prognosis predictable: number of teeth lost (T1–T4), upper/lower maxilla (U/L), alveolar/basal bone (Alv/B), gingival tissues (G), soft tissues (S), adult/child (a/c), and reconstructed patient (R) (Table 2).

For prosthetic rehabilitation, we placed dental implants from three different manufacturers: “3i Biomet," “BioHorizons Laser-Lock tapered,” and “Zimmer Trabecular Metal” implants.

All three kinds of implants have tapered body type and internal hex connection; the main differences are surface treatment that consists in three different technologies favoring osseointegration (NanoTite, Laser-Lock, and Trabecular Metal) and the tantalum composition of “Zimmer Trabecular Metal.”

3i Biomet NanoTite implant's surface maximizes the potential biological benefits of Calcium Phosphate (CaP).

Laser-Lok is a series of precision-engineered cell-sized channels laser-machined onto the surface of dental implants and abutments that allows physical connective tissue attachment.

Zimmer Biomet's Trabecular Metal Material is a highly porous biomaterial made from elemental tantalum with structural, functional, and physiological properties similar to those of bone. This material features an open, engineered, and interconnected pore structure to support bony in-growth and vascularization.

We divided the 50 patients in 3 different groups (group A, group B, and group C) based on the amount of tissue loss suffered.

All implants received a prosthesis after 3 months from healing screws insertion.

32 patients (group A) were affected by dentoalveolar bone loss due to low kinetic energy traumas as falls and assaults. Each patient of this group lost no more than 3 teeth (T1-T2) in the same site and basal bone was not involved in fracture.

18 received immediate implant insertion; 14 necessitated bone regeneration using particulate bone and collagen membranes contextually to implant positioning.

In 16 cases we did not need bone regeneration so we placed the implants after 1 month from site reclamation. We used 21 “3i Biomet,” 11 “BioHorizons Laser-Lock tapered,” and 36 “Zimmer Trabecular Metal” implants for a total of 68 implants positioned. In 2 cases of this group we needed bone grafts contextual to implant positioning. Healing screws were inserted at 4 months from implant positioning. All implants received a prosthesis after 3 months from healing screws insertion.

14 patients necessitated primary guided bone regeneration (GBR) using particulate “Zimmer Copios bone” and “Zimmer Collagen membranes” rebuilt after 2 months on average from site reclamation.

In 11 cases we performed implant insertion after primary bone reconstruction simultaneously to a further bone graft during implant surgery; in 3 cases bone reconstruction was made in two steps.

After bone reconstruction we waited 6 months on average before implant positioning; in the 3 cases with a two-step reconstruction we waited 1 year.

We placed 27 “3i Biomet,” 18 “BioHorizons Laser-Lock tapered,” and 11 “Zimmer Trabecular Metal” implants for a total of 56 implants positioned.

Healing screws were inserted at 4 months from implant positioning.

All group A patients have 1-year follow-up with no complications.

13 patients (group B) were affected by basal bone loss and required reconstruction surgery primary to implant positioning. Each patient of this group lost more than 3 teeth (T3, T4). 10 patients (B1) received bone graft taken from intraoral sites as mandibular angle, symphysis, and retromolar. The remaining 3 patients (B2) needed major quantity of bone: it was taken from iliac crest, skullcap, and fibula.

So we placed 11 “3i Biomet,” 9 “BioHorizons Laser-Lock tapered,” and 29 “Zimmer Trabecular Metal” implants for a total of 49 implants positioned.

Healing screws were inserted at 4 months from implant positioning.

All implants received a prosthesis after 3 months from healing screws insertion.

All group B patients have 1-year follow-up with no complications.

5 patients (group C) have lost soft tissues, big portions of basal bone, and more than 5 teeth (T4). We performed a flap revascularized fibula. These patients had the more difficult prosthetic rehabilitation due to the big loss of tissues. So we designed a maxillofacial prosthesis comprising a primary structure supported by implants and a secondary structure with aesthetic and functional characteristics.

So we placed 8 “3i Biomet,” 10 “BioHorizons Laser-Lock tapered,” and 16 “Zimmer Trabecular Metal” implants for a total of 34 implants positioned.

Healing screws were inserted at 4 months from implant positioning.

All implants received a prosthesis after 3 months from healing screws insertion.

All group C patients have 1-year follow-up with no complications.

Evaluation included assessment of implant survival, mucositis, and peri-implantitis. Measurements of bone level changes were made clinically and radiologically by 3 different operators of the department, by evaluating bone level mesially and distally to each implant at implant placement, 4, 6, 12 months later. We measured the vertical distance from the neck of the implant to the crest of the surrounding bone tissue to evaluate peri-implant bone loss.

Each implant inserted underwent clinical examination in 5 different times:

1. when entering with the execution of an intraoral X-ray and a torque control insertion;
2. after 4 months during healing screws insertion with the execution of an intraoral X-ray;
3. after 3 months from the inclusion of the healing screws with the execution of a rx intraoral and a peri-implant survey;
4. after further 6 months by performing an intraoral X-ray and a peri-implant survey;
5. last check 6 months later from the previous one with the aid of only peri-implant survey.

## 3. Results

### 3.1. Case  1: Group A

A 24-year-old male patient suffered a traumatic event using a circular saw.

**Figure 1:**
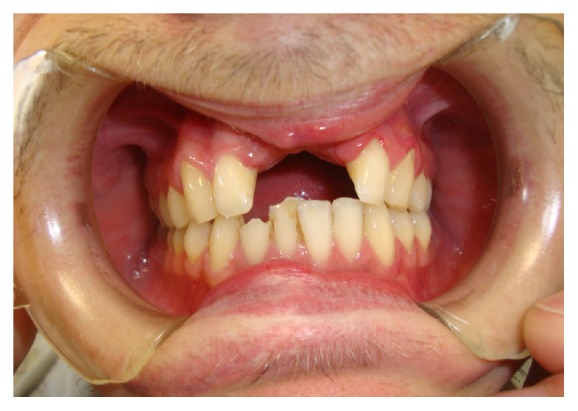


The injury caused the loss of teeth 1.1 and 2.1, and the loss of a portion of basal bone in premaxilla area, leaving an edentulous concave that extends 2 × 0,5 cm (Figure 1).

**Figure 2:**
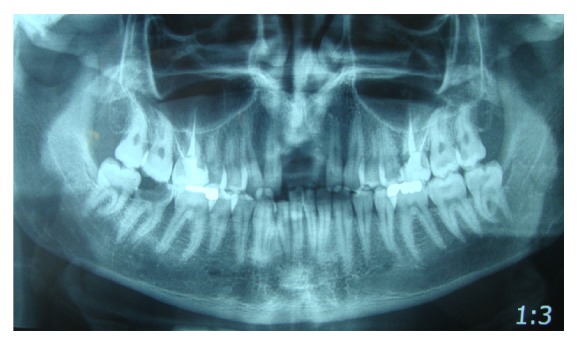


Rx orthopanoramic showed the extent and the type of bone loss, and so we required a CT cone-beam to study prosthetic rehabilitation (Figure 2).

The analysis of CT examination leads to the choice of the treatment plan, which includes the performance of a bone graft and 6 months later the insertion of two implants by “two-stage" technique.

Then a dental impression in alginate was taken to build a resin removable partial denture to rehabilitate the patient provisionally.

**Figure 3:**
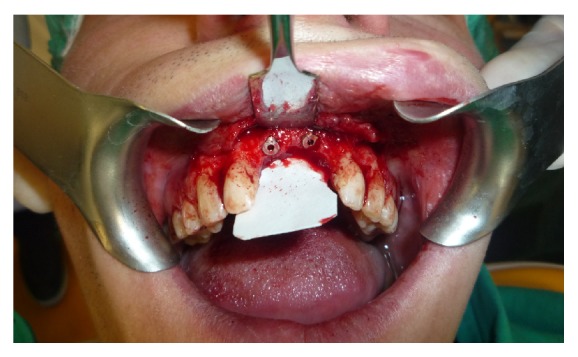


**Figure 4:**
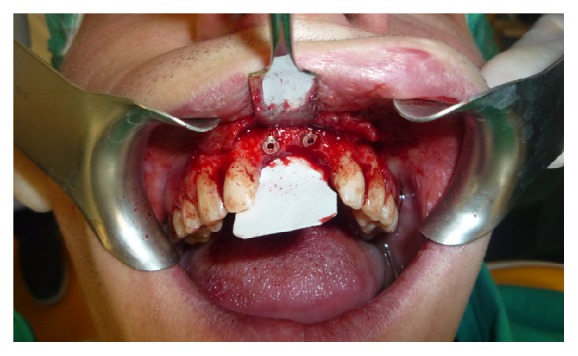


The regeneration is accomplished through the use of an allograft of bovine particulate bone (“Zimmer CopiOs bone”) and a resorbable collagen membrane 20 × 30 mm (“Zimmer Collagen membrane”), while in second surgery 2 implants 4.1 mm × 11.5 mm were placed with trabecular morphology and tantalum coated (“Zimmer Trabecular Metal”) (Figures 3 and 4).

Removable partial denture was modified to not load on inserted implants.

After 4 months healing screws were inserted, and 3 months later the patient was prosthesized by 2 zirconium crowns.

**Figure 5:**
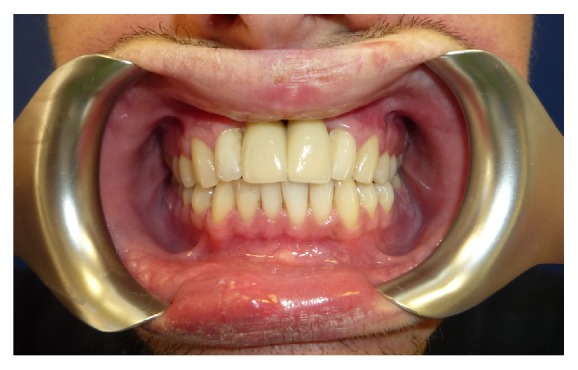


The entire dental treatment lasted 8 months; follow-up at one year was uneventful (Figure 5).

### 3.2. Case  2: Group B1

**Figure 6:**
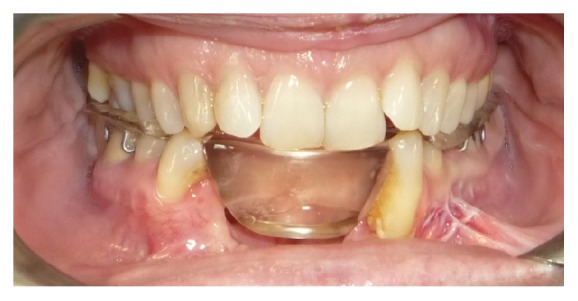


A 41-year-old female patient was wounded by a ballistic trauma that caused the loss of teeth 4.3, 4.2, 4.1, 3.1, and 3.2, the loss of a big portion of basal bone and gingiva in this area, and the loss of an eye (Figure 6).

At first, we took a dental impression in alginate to build a resin removable partial denture to rehabilitate the patient provisionally.

**Figure 7:**
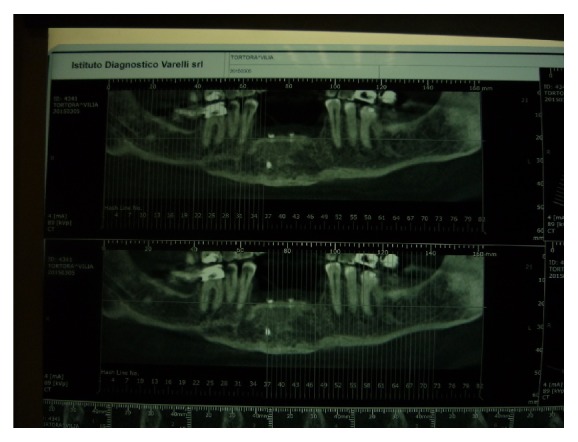


In collaboration with Maxillofacial Unit a treatment plan waiting 8 months from mandibular repositioning was scheduled which provided a surgical bone graft from intraoral sites and the subsequent implants placement (Figure 7).

**Figure 8:**
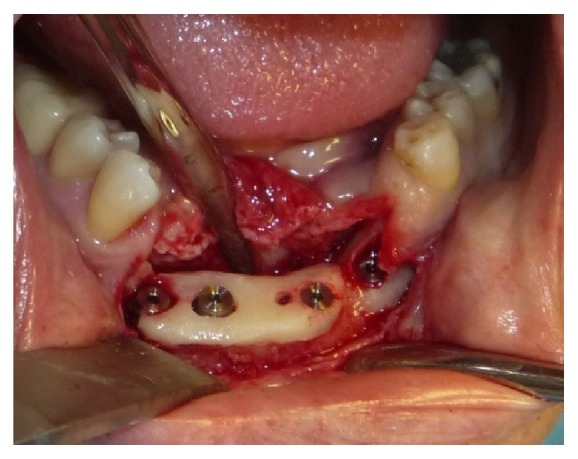


At first surgery bone regeneration was performed taking the bone from the mandibular angle. After 9 months we placed 2 implants 3.7 mm × 11.5 mm “Zimmer Trabecular Metal” and 2 implants 4.1 mm × 13 mm “Zimmer Trabecular Metal” (Figure 8).

Removable partial denture was modified to not load on inserted implants.

**Figure 9:**
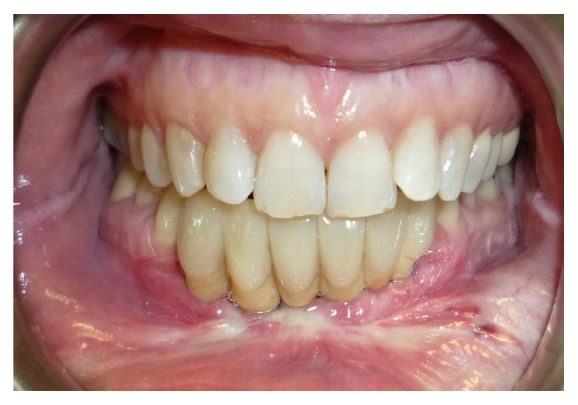


In the same area a fornix depth was performed using a conformer to earn attached gingiva. After 4 months healing screws were inserted, and 3 months later the patient was prosthesized by 5 metal-ceramic crowns (Figure 9).

The entire dental treatment lasted 14 months; follow-up at one year was uneventful.

### 3.3. Case  3: Group B2

A 23-year-old female patient suffered multiple facial fractures due to a fall from a great height.

At first aid, CT showed both maxillary sinus' anterior and medial wall fractures with concomitant hemosinus, compound fracture of the right orbital floor, fracture of mandibular symphysis with involvement of alveolar processes, compound fracture of the right mandibular condyle with medial displacement of the proximal fragment, fracture of the left and front side of hard palate with involvement of the anterior alveolar process, and fractures of nasal septum and bones.

After establishing vital functions, the patient was operated on; surgery included reduction and contention of all fractures and concomitant extraction of 3.1, 3.2, 3.3, 3,4, 4.1, 4.2, 4.3, 4.4, and 4.5.

**Figure 10:**
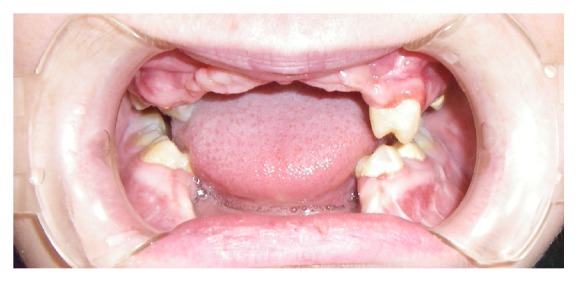


Nine months later the patient began prosthetic rehabilitation protocol; in fact she removed the restrains and surgery had success (Figure 10).

**Figure 11:**
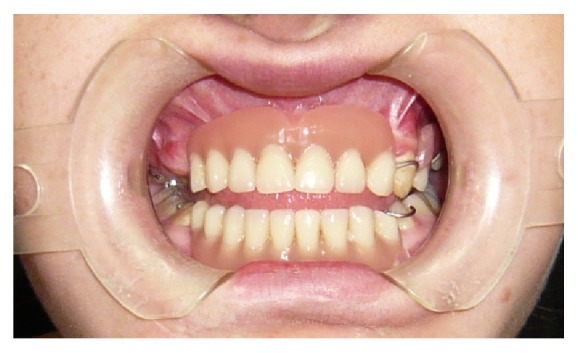


Then a dental impression was taken in alginate to build a resin removable partial denture to rehabilitate the patient provisionally (Figure 11).

A radiographic-surgery template was projected to give reference points to prosthodontist because the patient suffered a big loss of hard and soft tissue.

The CT cone-beam with template inserted showed us the quality and the quantity of bone directing choice of kind of implants and implants positioning.

The case study directed us to rehabilitate the patient by implant supported prosthesis consisting of 3 different components: a titanium base screwed on implants, a titanium structure (primary structure) assembled on the base, and a composite coated structure (secondary structure) that reproduced teeth and gum.

**Figure 12:**
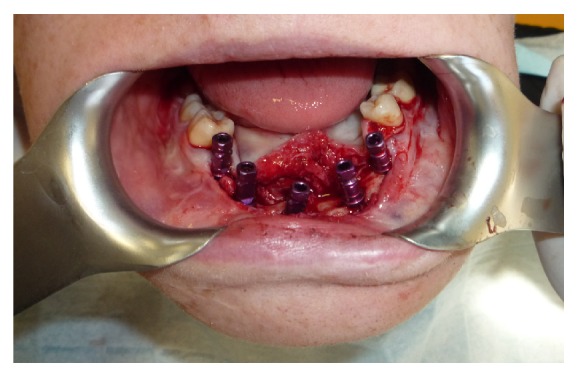


**Figure 13:**
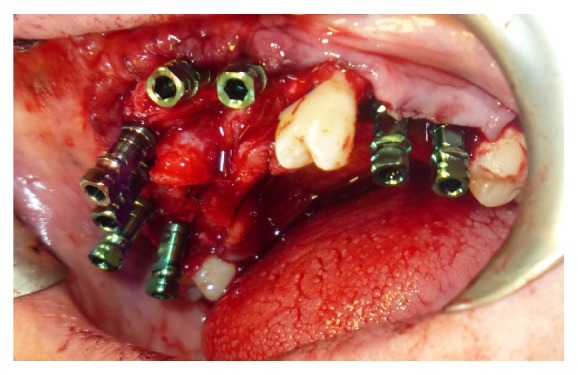


At first surgery we placed five implants “Zimmer Trabecular Metal” 4,7 × 11,5 mm in mandible and 8 implants “Zimmer Trabecular Metal” 4,1 × 10 mm in maxilla with simultaneous bone graft using “Zimmer CopiOs bone” and “Zimmer Collagen membrane” (Figures 12 and 13).

In mandible a fornix depth was performed using a conformer to earn attached gingiva.

**Figure 14:**
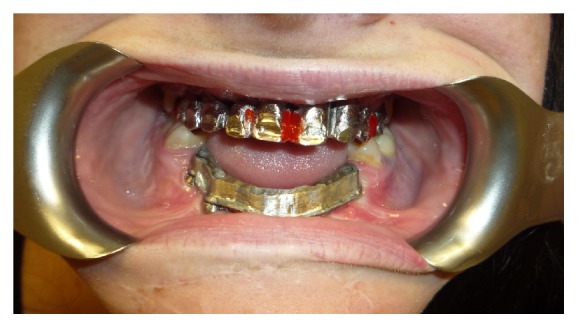


After 4 months we inserted healing screws and after 6 months we started testing metal structure and teeth (Figure 14).

**Figure 15:**
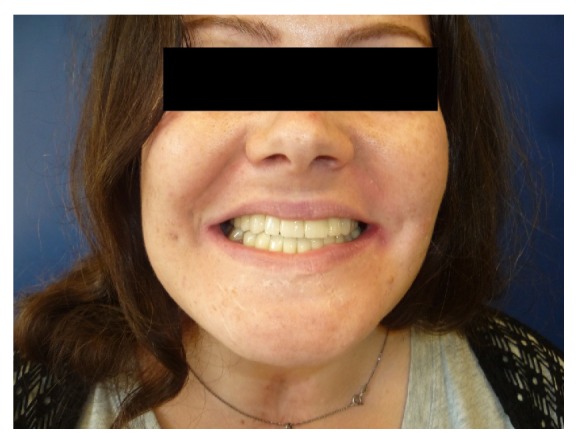


The entire dental treatment lasted 22 months; follow-up at one year was uneventful (Figure 15).

### 3.4. Case  4: Group C

A 29-year-old male patient was wounded by a ballistic trauma that caused the destruction of right premaxilla and of dental elements 1.1, 1.2, 1.3, 1.4, 1.5, and 2.1 and adjacent soft tissue.

**Figure 16:**
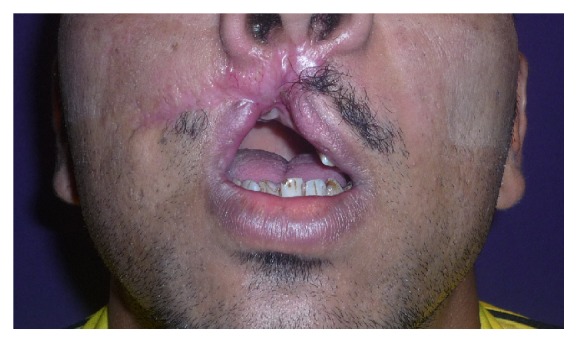


**Figure 17:**
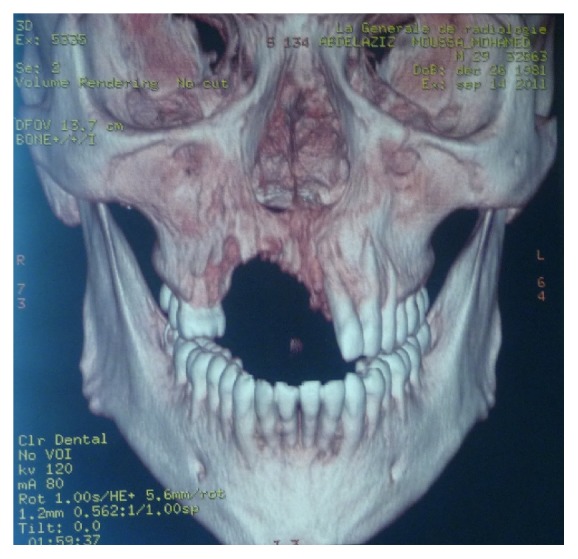


The patient lost a big portion of labial soft tissue and showed a retracting and hypertrophic scar in this zone (Figures 16 and 17).

At first surgery the reconstruction of the area was performed by osteomyocutaneous fibula free flap. Four months later the patient began prosthetic rehabilitation by implant supported prosthesis consisting of 3 different components: a titanium base screwed on implants, a titanium structure (primary structure) assembled on the base, and a composite coated structure (secondary structure) that reproduced teeth and gingiva.

Then a dental impression was taken in alginate to build a resin removable partial denture to rehabilitate the patient provisionally.

A radiographic-surgery template was projected to give reference points to prosthodontist because the patient suffered a big loss of hard and soft tissue due to the injury.

At second surgery we placed six implants “Zimmer Trabecular Metal” 4.1 × 10 mm in dental element loss position.

**Figure 18:**
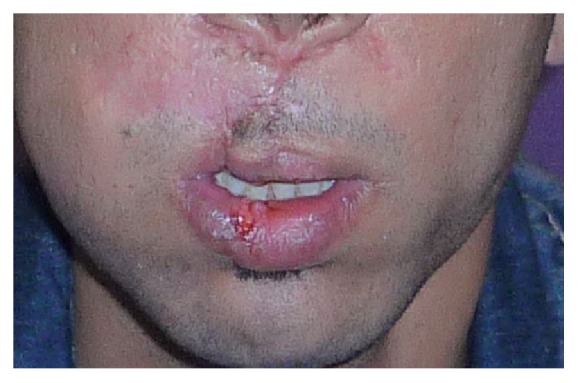


In a second step, the reconstruction of the upper lip using an Abbè mucocutaneous flap was performed (Figure 18).

After 4 months we inserted healing screws and after 6 months we started testing metal structure and teeth.

**Figure 19:**
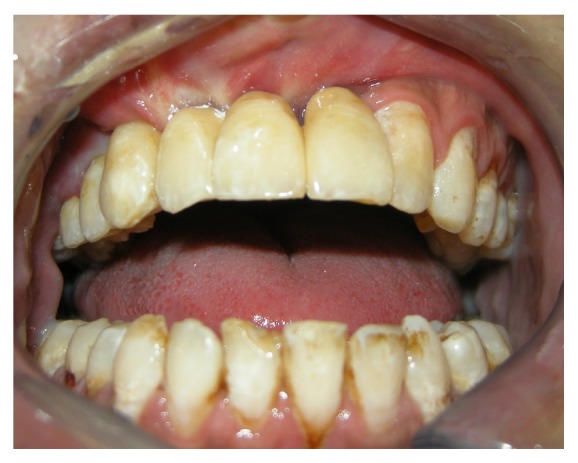


The entire dental treatment lasted 14 months; follow-up at one year was uneventful (Figure 19).

### 3.5. Clinical Evaluation

Cumulative implant survival rate in all groups (A, B1, B2, and C) is 97,1% (*n* = 201/207) to date and all implants had at least 12 months of clinical follow-up after functional loading.

6 implants (1 in group B1, 2 in group B2, and 3 in group C) were loss due to peri-implantitis.

Mean crestal marginal bone loss was 0.17 ± 0.25 mm after 2 months of functional loading on periapical radiographs, 0.22 ± 0.4 mm at 4 months, 0.3 ± 0.46 at 6 months, and 0.58 ± 0.62 at 1 year.

Implant stability was evaluated by Periotest values at 6 months. The mean Periotest value and Standard Deviation for implant at 6 months were −2.15 ± 1.19 (group A), −2.21 ± 1.57 (group B1), −2.29 ± 1.70 (group B2), and −1.50 ± 1.62 (group C).

## 4. Discussion

During the treatment of the traumatized patients, prosthodontist finds a lot of variable pathologic situations that involve other medical specialties as maxillofacial surgery, plastic surgery, emergency surgery, otolaryngology, physiotherapy, speech therapy, orthopedics, and ophthalmology.

The multidisciplinary approach and the collaboration between multiple clinical figures are therefore critical for the success of the treatment of these patients.

The purpose of this study is to make predictable patients outcomes classifying their clinical data, using certain parameters and introducing a new classification method.

The decision to introduce a new classification comes from the complete separation in actual classifications between dental trauma and facial trauma, except Andreasen's classification [12].

Comparing and accumulating data from different studies is extremely difficult due to the differences in the definitions and classifications used [13].

Andreasen's classification represents the most complete classification containing 19 groups and includes injuries to the teeth, supporting structures, gingiva, and oral mucosa but does not include facial and rehabilitation features. It is a modification of World Heal Organization's (WHO) classification of dental trauma [14] that includes only injuries to the teeth and contains a group named “other injuries including laceration of oral soft tissues” that is misleading for investigating purposes.

Ellis' classification [15] and Garcia-Godoy's classification [16] are other modifications of WHO classification of dental trauma that also do not have groups about alveolar, maxilla, or mandibular trauma.

The most commonly used classification for describing facial fractures remains that classically described by LeFort [17], which alone yields insufficient information for fracture description and the complete planning of treatment [18].

Other classifications were described to supplement the LeFort description and were based on detailed descriptions of fractures of individual midfacial regions, such as orbitozygomatic fractures classified by Zingg et al. [19] and the nasoethmoid classification by Leipziger and Manson [20, 21].

Unfortunately all these classifications do not consider oral tissues and dental involvement.

In our classification proposal the presence and quantification of above parameters influence the treatment protocol; patients undergo different levels of treatment depending on the measured data.

The etiology of the trauma has a significant influence on clinical parameters; serious road accidents, falls from great heights, and ballistic trauma by firearms are the etiologic categories in which patients are more difficult to treat.

Our clinical experience allowed the formulation of this indexing to help physician to make predictable patients outcomes: common parameters of reference permit a better disease framing to treat patients strategically.

Treatment is influenced by the entity and by the presence of these clinical parameters: a greater number of lost teeth (T) require more time for prosthetic rehabilitation; basal bone damage (B) involves a lack of support for implants placement that need a bone graft or a reconstructive surgery; gingival tissue (G) could need a periodontal surgery intervention; soft tissue damage (S) could require a plastic surgery; and finally reconstructed patients (R) involve multidisciplinary approach and are more difficult to rehabilitate.

## 5. Conclusion

Facial traumas necessitate the collaboration between many clinical figures as maxillofacial surgeon, plastic surgeon, and prosthodontist. The multidisciplinary approach is helped by a painstaking clinical data collection. The recognition of certain clinical parameters is fundamental to frame diagnosis and successful treatment planning. Patients suffering soft tissues damage and reconstructed patients are the most difficult to rehabilitate. Predictability of patients outcomes is the key to better plan traumatized patients. Soft tissues represent a subjective element of evaluation that can alter our parameters.

## Figures and Tables

**Table 1:** 

Groups	Classification	Subgroups	Treatment
A (32)	T1-T2; Alv	—	Implants insertion +− contextual bone regeneration
B (13)	T3-T4; AlvB	B1 (10)	Bone graft from intraoral sites + implants insertion
		B2 (3)	Bone graft from other sites + implants insertion
C (5)	T3-T4; AlvB; S	—	Flap + implants insertion

**Table 2:** 

Parameters	Meaning	Classification
T	Number of teeth lost	T1 <2; T2 2-3; T3 4-5; T4 >5
U/L	Upper/lower maxilla	U; L; UL
Alv/B	Alveolar/basal bone	Alv; B; AlvB
G	Gingival tissue	G
S	Soft tissue	S
a/c	Adult/child	a; c
R	Reconstructed patient	R
